# (2-Hy­droxy-7-meth­oxy­naphthalen-1-yl)(phen­yl)methanone

**DOI:** 10.1107/S1600536810038547

**Published:** 2010-09-30

**Authors:** Atsushi Nagasawa, Ryosuke Mitsui, Yuichi Kato, Akiko Okamoto, Noriyuki Yonezawa

**Affiliations:** a Department of Organic and Polymer Materials Chemistry, Tokyo University of Agriculture & Technology, 2-24-16 Naka-machi, Koganei, Tokyo 184-8588, Japan

## Abstract

In the mol­ecule of the title compound, C_18_H_14_O_3_, there is an intra­molecular O—H⋯O=C hydrogen bond between the carbonyl and hy­droxy groups on the naphthalene ring system. The angles between the C=O bond vector and the least-squares planes of the naphthalene ring system and the phenyl ring are 30.58 (6) and 42.82 (7)°, respectively, while the dihedral angle between the naphthalene ring system and the phenyl ring is 58.65 (5)°. In the crystal, mol­ecules are connected by pairs of inter­molecular O—H⋯O=C hydrogen bonds, forming centrosymmetric dimers.

## Related literature

For closely related structures, see: Hijikata *et al.* (2010 ▶); Kato *et al.* (2010 ▶); Mitsui *et al.* (2009 ▶); Mitsui, Nakaema, Noguchi, Okamoto & Yonezawa (2008 ▶); Mitsui, Nakaema, Noguchi & Yonezawa (2008 ▶).
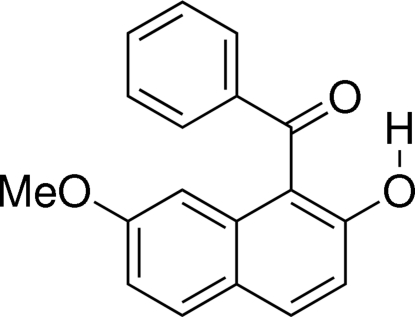


## Experimental

### 

#### Crystal data

C_18_H_14_O_3_
*M* *_r_* = 278.29
Monoclinic, 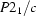

*a* = 9.81012 (18) Å
*b* = 6.27891 (11) Å
*c* = 22.0737 (4) Å
β = 93.167 (1)°
*V* = 1357.59 (4) Å^3^
*Z* = 4
Cu *K*α radiation
μ = 0.75 mm^−1^
*T* = 193 K
0.60 × 0.40 × 0.40 mm

#### Data collection

Rigaku R-AXIS RAPID diffractometer
Absorption correction: numerical (*NUMABS*; Higashi, 1999 ▶) *T*_min_ = 0.586, *T*_max_ = 0.754
20565 measured reflections
2496 independent reflections
2244 reflections with *I* > 2σ(*I*)
*R*_int_ = 0.038

#### Refinement

*R*[*F* ^2^ > 2σ(*F* ^2^)] = 0.036
*wR*(*F* ^2^) = 0.103
*S* = 1.08
2496 reflections
196 parameters
H atoms treated by a mixture of independent and constrained refinement
Δρ_max_ = 0.23 e Å^−3^
Δρ_min_ = −0.16 e Å^−3^

### 

Data collection: *PROCESS-AUTO* (Rigaku, 1998 ▶); cell refinement: *PROCESS-AUTO*; data reduction: *CrystalStructure* (Rigaku/MSC, 2004 ▶); program(s) used to solve structure: *SIR2004* (Burla *et al.*, 2005 ▶); program(s) used to refine structure: *SHELXL97* (Sheldrick, 2008 ▶); molecular graphics: *ORTEPIII* (Burnett & Johnson, 1996 ▶); software used to prepare material for publication: *SHELXL97*.

## Supplementary Material

Crystal structure: contains datablocks global, I. DOI: 10.1107/S1600536810038547/is2606sup1.cif

Structure factors: contains datablocks I. DOI: 10.1107/S1600536810038547/is2606Isup2.hkl

Additional supplementary materials:  crystallographic information; 3D view; checkCIF report

## Figures and Tables

**Table 1:** Hydrogen-bond geometry (Å, °)

*D*—H⋯*A*	*D*—H	H⋯*A*	*D*⋯*A*	*D*—H⋯*A*
O1—H1⋯O3	0.92 (2)	1.77 (2)	2.5792 (14)	145 (2)
O1—H1⋯O3^i^	0.92 (2)	2.32 (2)	3.0088 (16)	132.4 (18)

Symmetry code: (i) 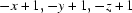.

**Fig. 1.**
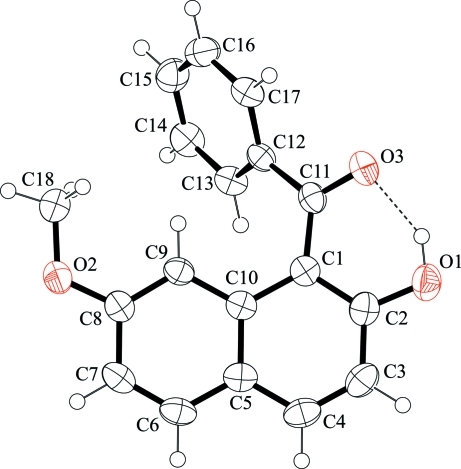
The asymmetric unit of compound (I), showing 50% probability displacement ellipsoids. The dashed line indicates an intramolecular O—H···O hydrogen bond.

**Fig. 2.**
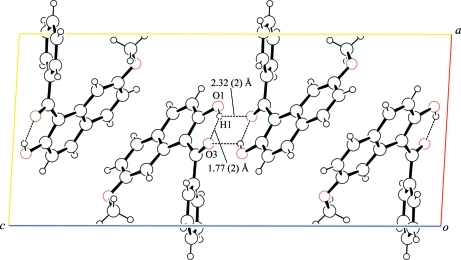
A partial crystal packing diagram of compound (I), viewed down the b axis. The dashed lines indicate intra- and intermolecular O—H···O hydrogen bonds.

## supplementary crystallographic information

### Comment

Recently, we reported the crystal structures of several 1-aroylated 2,7-dimethoxynaphthalene homologues exemplified by 1-benzoyl-2,7-dimethoxynaphthalene (Kato *et al.*, 2010) and 1-(4-chlorobenzoyl)-2,7-dimethoxynaphthalene (Mitsui, Nakaema, Noguchi, Okamoto & Yonezawa, 2008). Methyl 4-(2,7-dimethoxy-1-naphthoyl)benzoate (Hijikata *et al.*, 2010). Furthermore, we also reported the crystal structure of 1-monoaroylnaphthalene derivatives having 2-oxy group exemplified by (4-chlorobenzoyl)(2-hydroxy-7-methoxynaphthalene-1-yl)metanone (Mitsui, Nakaema, Noguchi & Yonezawa, 2008) and (4-chlorophenyl)(2-ethoxy-7-methoxynaphthalen-1-yl)methanone (Mitsui *et al.*, 2009). As a part of our ongoing studies on the synthesis and crystal structure analysis of aroylated naphthalene derivatives, we prepared and analysed the crystal structure of 1-benzoyl-2-hydroxy-7-methoxynaphthalene (I). The title compound was prepared by chemoselective demethylation of 1-benzoyl-2,7-dimethoxynaphthalene with aluminium trichloride.

An *ORTEPIII* (Burnett & Johnson, 1996) plot of (I) is shown in Fig. 1. In the molecule of (I), the intramolecular O—H···O═C hydrogen bond that forms a six-membered ring including carbonyl and hydroxy groups on the naphthalene ring is observed [O3···H1 = 1.77 (2) Å]. The conformation of these groups resembles to that of (4-chlorobenzoyl)(2-hydroxy-7-methoxynaphthalen-1-yl)metanone (Mitsui, Nakaema, Noguchi & Yonezawa, 2008). The angles of C═O bond vector against the least-squares plane of the naphthalene ring (C1–C10) and benzene ring (C12–C17) are 30.58 (6) and 42.82 (7)°, respectively. The dihedral angle between the naphthalene ring (C1–C10) and benzene ring (C12–C17) is 58.65 (5)°.

In the crystal structure, the molecular packing of (I) is mainly stabilized by intermolecular hydrogen bond and van der Waals interaction. Two adjacent naphthalene rings are exactly parallel and the intermolecular O—H···O═C hydrogen bond between the hydroxy group and the carbonyl oxygen on the naphthalene ring (Fig. 2) along the *c* axis, is observed [O3···H1 = 2.32 (2) Å]. The oxygen atom in the methoxy group interacts with carbon atom in the methoxy group of the next molecule, *i.e.* two methoxy groups in the adjacent molecules interact with each other [O2···C18 = 3.060 (2) Å] along the *a* axis. The naphthalene rings interact with the carbonyl groups [C4···O3 = 3.036 (18) Å] along the *b* axis. The benzoyl groups interact with the methyl groups (C16···H18A = 2.88 Å) along the *a* axis.

### Experimental

To a solution of 1-benzoyl-2,7-dimethoxynaphthalene (2.92 g, 10 mmol) in CH_2_Cl_2_ (100 ml) was added AlCl_3_ (6.65 g, 50 mmol). The reaction mixture was refluxed for 30 min giving a dark red solution, which was then poured into H_2_O (30 ml). The aqueous layer was extracted with CHCl_3 _(30 ml × 3). The combined organic layers were washed with brine (30 ml × 3), and dried over MgSO_4_ overnight. The solvent was removed *in vacuo* and the crude material was purified by recrystallization from hexane to give compound (I) as yellow platelets (m.p. 371.8–372.3 K, yield 1.45 g, 52%).

Spectroscopic Data: ^1^H NMR (300 MHz, CDCl_3_) δ 11.64 (s, 1H), 7.85 (d, 1H), 7.64–7.60 (m, 3H), 7.55 (tt, 1H), 7.43 (t, 2H) 7.08 (d, 1H), 6.89 (dd, 1H), 6.59 (d, 1H), 3.27 (s, 3H); ^13^C NMR (75 MHz, CDCl_3_) δ 200.8, 162.8, 158.2, 140.8, 136.5, 134.1, 132.3, 130.1, 129.14, 128.8, 123.7, 116.5, 115.9, 113.7, 106.5, 54.5; IR (KBr): 3446, 1617, 1572, 1511, 1200; HRMS (*m/z*): [*M* + H]^+^ calcd for C_18_H_15_O_3_, 279.1021; found, 279.0999.

### Refinement

All the H-atoms could be located in difference Fourier maps. The OH hydrogen atom was freely refined: O1—H1 = 0.92 (2) Å. The C-bound H-atoms were subsequently refined as riding atoms, with C—H = 0.95 (aromatic) and 0.98 (methyl) Å, and with *U*_iso_(H) = 1.2*U*_eq_(C).

### Crystal data

**Table:**

C_18_H_14_O_3_	*F*(000) = 584
*M**_r_* = 278.29	*D*_x_ = 1.362 Mg m^−^^3^
Monoclinic, *P*2_1_/*c*	Melting point = 371.8–372.3 K
Hall symbol: -P 2ybc	Cu *K*α radiation, λ = 1.54187 Å
*a* = 9.81012 (18) Å	Cell parameters from 19252 reflections
*b* = 6.27891 (11) Å	θ = 4.0–68.2°
*c* = 22.0737 (4) Å	µ = 0.75 mm^−^^1^
β = 93.167 (1)°	*T* = 193 K
*V* = 1357.59 (4) Å^3^	Block, yellow
*Z* = 4	0.60 × 0.40 × 0.40 mm

### Data collection

**Table:**

Rigaku R-AXIS RAPID diffractometer	2496 independent reflections
Radiation source: rotating anode	2244 reflections with *I* > 2σ(*I*)
graphite	*R*_int_ = 0.038
Detector resolution: 10.00 pixels mm^-1^	θ_max_ = 68.2°, θ_min_ = 4.0°
ω scans	*h* = −11→11
Absorption correction: numerical (*NUMABS*; Higashi, 1999)	*k* = −7→7
*T*_min_ = 0.586, *T*_max_ = 0.754	*l* = −26→26
20565 measured reflections	

### Refinement

**Table:**

Refinement on *F*^2^	Secondary atom site location: difference Fourier map
Least-squares matrix: full	Hydrogen site location: inferred from neighbouring sites
*R*[*F*^2^ > 2σ(*F*^2^)] = 0.036	H atoms treated by a mixture of independent and constrained refinement
*wR*(*F*^2^) = 0.103	*w* = 1/[σ^2^(*F*_o_^2^) + (0.0535*P*)^2^ + 0.3346*P*] where *P* = (*F*_o_^2^ + 2*F*_c_^2^)/3
*S* = 1.08	(Δ/σ)_max_ < 0.001
2496 reflections	Δρ_max_ = 0.23 e Å^−^^3^
196 parameters	Δρ_min_ = −0.16 e Å^−^^3^
0 restraints	Extinction correction: *SHELXL97* (Sheldrick, 2008), Fc^*^=kFc[1+0.001xFc^2^λ^3^/sin(2θ)]^-1/4^
Primary atom site location: structure-invariant direct methods	Extinction coefficient: 0.0146 (8)

### Special details

**Table:**

Geometry. All e.s.d.'s (except the e.s.d. in the dihedral angle between two l.s. planes) are estimated using the full covariance matrix. The cell e.s.d.'s are taken into account individually in the estimation of e.s.d.'s in distances, angles and torsion angles; correlations between e.s.d.'s in cell parameters are only used when they are defined by crystal symmetry. An approximate (isotropic) treatment of cell e.s.d.'s is used for estimating e.s.d.'s involving l.s. planes.
Refinement. Refinement of *F*^2^ against ALL reflections. The weighted *R*-factor *wR* and goodness of fit *S* are based on *F*^2^, conventional *R*-factors *R* are based on *F*, with *F* set to zero for negative *F*^2^. The threshold expression of *F*^2^ > σ(*F*^2^) is used only for calculating *R*-factors(gt) *etc*. and is not relevant to the choice of reflections for refinement. *R*-factors based on *F*^2^ are statistically about twice as large as those based on *F*, and *R*- factors based on ALL data will be even larger.

### Fractional atomic coordinates and isotropic or equivalent isotropic displacement parameters (Å^2^)

**Table:**

	*x*	*y*	*z*	*U*_iso_*/*U*_eq_	
O1	0.37752 (10)	0.17334 (19)	0.46905 (5)	0.0478 (3)	
O2	0.84529 (10)	−0.02213 (17)	0.22739 (4)	0.0442 (3)	
O3	0.57091 (10)	0.44133 (17)	0.45195 (5)	0.0482 (3)	
C1	0.55482 (12)	0.1185 (2)	0.39823 (5)	0.0336 (3)	
C2	0.43388 (13)	0.0609 (2)	0.42511 (6)	0.0387 (3)	
C3	0.36518 (14)	−0.1302 (3)	0.40877 (7)	0.0451 (4)	
H3	0.2846	−0.1684	0.4281	0.054*	
C4	0.41359 (14)	−0.2593 (2)	0.36562 (7)	0.0431 (3)	
H4	0.3685	−0.3902	0.3564	0.052*	
C5	0.53019 (13)	−0.2030 (2)	0.33398 (6)	0.0363 (3)	
C6	0.57599 (14)	−0.3323 (2)	0.28681 (6)	0.0407 (3)	
H6	0.5321	−0.4647	0.2784	0.049*	
C7	0.68125 (14)	−0.2718 (2)	0.25323 (6)	0.0401 (3)	
H7	0.7113	−0.3612	0.2219	0.048*	
C8	0.74557 (13)	−0.0737 (2)	0.26554 (6)	0.0356 (3)	
C9	0.70639 (13)	0.0537 (2)	0.31195 (6)	0.0335 (3)	
H9	0.7512	0.1860	0.3194	0.040*	
C10	0.59982 (12)	−0.0095 (2)	0.34895 (5)	0.0322 (3)	
C11	0.63086 (13)	0.3022 (2)	0.42425 (5)	0.0341 (3)	
C12	0.78243 (13)	0.3247 (2)	0.42242 (5)	0.0327 (3)	
C13	0.87010 (14)	0.1539 (2)	0.43450 (6)	0.0383 (3)	
H13	0.8342	0.0163	0.4417	0.046*	
C14	1.01009 (14)	0.1857 (2)	0.43590 (7)	0.0443 (4)	
H14	1.0701	0.0698	0.4448	0.053*	
C15	1.06297 (14)	0.3852 (3)	0.42446 (7)	0.0469 (4)	
H15	1.1590	0.4054	0.4246	0.056*	
C16	0.97576 (15)	0.5548 (2)	0.41281 (7)	0.0456 (4)	
H16	1.0119	0.6917	0.4049	0.055*	
C17	0.83608 (14)	0.5257 (2)	0.41259 (6)	0.0392 (3)	
H17	0.7766	0.6437	0.4057	0.047*	
C18	0.92146 (16)	0.1669 (3)	0.24071 (8)	0.0527 (4)	
H18A	0.9894	0.1867	0.2103	0.063*	
H18B	0.9679	0.1545	0.2810	0.063*	
H18C	0.8595	0.2894	0.2400	0.063*	
H1	0.428 (2)	0.296 (4)	0.4744 (11)	0.092 (8)*	

### Atomic displacement parameters (Å^2^)

**Table:**

	*U*^11^	*U*^22^	*U*^33^	*U*^12^	*U*^13^	*U*^23^
O1	0.0369 (5)	0.0601 (7)	0.0471 (6)	0.0032 (5)	0.0080 (4)	−0.0043 (5)
O2	0.0435 (5)	0.0478 (6)	0.0420 (5)	0.0003 (4)	0.0081 (4)	−0.0093 (4)
O3	0.0490 (6)	0.0453 (6)	0.0514 (6)	0.0042 (5)	0.0132 (5)	−0.0117 (5)
C1	0.0324 (6)	0.0356 (7)	0.0322 (6)	0.0041 (5)	−0.0032 (5)	0.0025 (5)
C2	0.0322 (6)	0.0468 (8)	0.0367 (7)	0.0046 (6)	−0.0011 (5)	0.0032 (6)
C3	0.0328 (7)	0.0543 (9)	0.0480 (8)	−0.0039 (6)	0.0004 (6)	0.0071 (7)
C4	0.0377 (7)	0.0415 (8)	0.0489 (8)	−0.0063 (6)	−0.0083 (6)	0.0064 (6)
C5	0.0349 (6)	0.0344 (7)	0.0384 (7)	0.0014 (5)	−0.0089 (5)	0.0028 (5)
C6	0.0409 (7)	0.0329 (7)	0.0466 (8)	−0.0003 (6)	−0.0116 (6)	−0.0035 (6)
C7	0.0422 (7)	0.0368 (7)	0.0402 (7)	0.0069 (6)	−0.0075 (6)	−0.0092 (6)
C8	0.0334 (6)	0.0389 (7)	0.0340 (6)	0.0055 (5)	−0.0030 (5)	−0.0017 (5)
C9	0.0340 (6)	0.0319 (7)	0.0340 (6)	0.0009 (5)	−0.0038 (5)	−0.0022 (5)
C10	0.0309 (6)	0.0327 (7)	0.0322 (6)	0.0041 (5)	−0.0061 (5)	0.0021 (5)
C11	0.0396 (7)	0.0343 (7)	0.0286 (6)	0.0055 (5)	0.0023 (5)	0.0011 (5)
C12	0.0376 (7)	0.0337 (7)	0.0265 (6)	0.0011 (5)	−0.0024 (5)	−0.0046 (5)
C13	0.0421 (7)	0.0337 (7)	0.0385 (7)	0.0010 (6)	−0.0040 (5)	−0.0017 (6)
C14	0.0406 (7)	0.0465 (8)	0.0449 (8)	0.0083 (6)	−0.0063 (6)	−0.0019 (6)
C15	0.0367 (7)	0.0582 (9)	0.0454 (8)	−0.0040 (7)	−0.0014 (6)	−0.0033 (7)
C16	0.0480 (8)	0.0428 (8)	0.0457 (8)	−0.0097 (6)	0.0001 (6)	0.0011 (6)
C17	0.0458 (8)	0.0333 (7)	0.0381 (7)	0.0025 (6)	−0.0031 (6)	−0.0023 (6)
C18	0.0512 (9)	0.0552 (10)	0.0531 (9)	−0.0099 (7)	0.0163 (7)	−0.0085 (7)

### Geometric parameters (Å, °)

**Table:**

O1—C2	1.3434 (17)	C8—C9	1.3716 (18)
O1—H1	0.92 (2)	C9—C10	1.4184 (18)
O2—C8	1.3646 (16)	C9—H9	0.9500
O2—C18	1.4244 (18)	C11—C12	1.4964 (18)
O3—C11	1.2340 (16)	C12—C17	1.3895 (19)
C1—C2	1.4029 (18)	C12—C13	1.3908 (18)
C1—C10	1.4414 (18)	C13—C14	1.386 (2)
C1—C11	1.4728 (18)	C13—H13	0.9500
C2—C3	1.413 (2)	C14—C15	1.384 (2)
C3—C4	1.357 (2)	C14—H14	0.9500
C3—H3	0.9500	C15—C16	1.381 (2)
C4—C5	1.418 (2)	C15—H15	0.9500
C4—H4	0.9500	C16—C17	1.382 (2)
C5—C6	1.413 (2)	C16—H16	0.9500
C5—C10	1.4233 (18)	C17—H17	0.9500
C6—C7	1.358 (2)	C18—H18A	0.9800
C6—H6	0.9500	C18—H18B	0.9800
C7—C8	1.4138 (19)	C18—H18C	0.9800
C7—H7	0.9500		
			
C2—O1—H1	107.2 (15)	C9—C10—C1	123.00 (12)
C8—O2—C18	117.15 (11)	C5—C10—C1	119.21 (11)
C2—C1—C10	118.47 (12)	O3—C11—C1	120.18 (12)
C2—C1—C11	117.33 (12)	O3—C11—C12	116.62 (12)
C10—C1—C11	124.15 (11)	C1—C11—C12	123.10 (11)
O1—C2—C1	124.13 (13)	C17—C12—C13	119.65 (12)
O1—C2—C3	114.90 (12)	C17—C12—C11	118.39 (11)
C1—C2—C3	120.91 (13)	C13—C12—C11	121.80 (12)
C4—C3—C2	120.41 (13)	C14—C13—C12	119.65 (13)
C4—C3—H3	119.8	C14—C13—H13	120.2
C2—C3—H3	119.8	C12—C13—H13	120.2
C3—C4—C5	121.32 (13)	C15—C14—C13	120.45 (13)
C3—C4—H4	119.3	C15—C14—H14	119.8
C5—C4—H4	119.3	C13—C14—H14	119.8
C6—C5—C4	121.16 (13)	C16—C15—C14	119.80 (13)
C6—C5—C10	119.56 (12)	C16—C15—H15	120.1
C4—C5—C10	119.26 (13)	C14—C15—H15	120.1
C7—C6—C5	121.57 (13)	C15—C16—C17	120.20 (14)
C7—C6—H6	119.2	C15—C16—H16	119.9
C5—C6—H6	119.2	C17—C16—H16	119.9
C6—C7—C8	119.07 (13)	C16—C17—C12	120.21 (13)
C6—C7—H7	120.5	C16—C17—H17	119.9
C8—C7—H7	120.5	C12—C17—H17	119.9
O2—C8—C9	124.22 (12)	O2—C18—H18A	109.5
O2—C8—C7	114.70 (12)	O2—C18—H18B	109.5
C9—C8—C7	121.07 (12)	H18A—C18—H18B	109.5
C8—C9—C10	120.84 (12)	O2—C18—H18C	109.5
C8—C9—H9	119.6	H18A—C18—H18C	109.5
C10—C9—H9	119.6	H18B—C18—H18C	109.5
C9—C10—C5	117.70 (12)		
			
C10—C1—C2—O1	−176.30 (12)	C6—C5—C10—C1	−178.29 (11)
C11—C1—C2—O1	6.22 (19)	C4—C5—C10—C1	3.48 (18)
C10—C1—C2—C3	6.53 (19)	C2—C1—C10—C9	168.90 (12)
C11—C1—C2—C3	−170.95 (12)	C11—C1—C10—C9	−13.80 (19)
O1—C2—C3—C4	−178.91 (13)	C2—C1—C10—C5	−7.45 (17)
C1—C2—C3—C4	−1.5 (2)	C11—C1—C10—C5	169.85 (11)
C2—C3—C4—C5	−2.7 (2)	C2—C1—C11—O3	−25.19 (18)
C3—C4—C5—C6	−176.55 (13)	C10—C1—C11—O3	157.48 (12)
C3—C4—C5—C10	1.7 (2)	C2—C1—C11—C12	150.82 (12)
C4—C5—C6—C7	175.00 (13)	C10—C1—C11—C12	−26.50 (18)
C10—C5—C6—C7	−3.20 (19)	O3—C11—C12—C17	−42.07 (17)
C5—C6—C7—C8	−0.6 (2)	C1—C11—C12—C17	141.79 (12)
C18—O2—C8—C9	6.22 (19)	O3—C11—C12—C13	133.31 (13)
C18—O2—C8—C7	−174.85 (12)	C1—C11—C12—C13	−42.84 (18)
C6—C7—C8—O2	−176.72 (12)	C17—C12—C13—C14	−0.74 (19)
C6—C7—C8—C9	2.25 (19)	C11—C12—C13—C14	−176.06 (12)
O2—C8—C9—C10	178.76 (11)	C12—C13—C14—C15	−1.0 (2)
C7—C8—C9—C10	−0.11 (19)	C13—C14—C15—C16	1.4 (2)
C8—C9—C10—C5	−3.58 (18)	C14—C15—C16—C17	0.0 (2)
C8—C9—C10—C1	−179.98 (11)	C15—C16—C17—C12	−1.8 (2)
C6—C5—C10—C9	5.17 (17)	C13—C12—C17—C16	2.1 (2)
C4—C5—C10—C9	−173.06 (12)	C11—C12—C17—C16	177.61 (11)

### Hydrogen-bond geometry (Å, °)

**Table:**

*D*—H···*A*	*D*—H	H···*A*	*D*···*A*	*D*—H···*A*
O1—H1···O3	0.92 (2)	1.77 (2)	2.5792 (14)	145 (2)
O1—H1···O3^i^	0.92 (2)	2.32 (2)	3.0088 (16)	132.4 (18)

Symmetry codes: (i) −*x*+1, −*y*+1, −*z*+1.
